# The Relative Age Effect in under-18 basketball: Effects on performance according to playing position

**DOI:** 10.1371/journal.pone.0200408

**Published:** 2018-07-09

**Authors:** Sergio J. Ibáñez, Aitor Mazo, Juarez Nascimento, Javier García-Rubio

**Affiliations:** 1 Faculty of Sports Sciences, University of Extremadura, Cáceres, Spain; 2 Universidade Federal de Santa Catarina, Florianópolis, Brasil; 3 Facultad de Educación, Universidad Autónoma de Chile, Santiago de Chile, Chile; Leeds Beckett University, UNITED KINGDOM

## Abstract

The Relative Age Effect (RAE) in sport is defined as the age difference in the same sport group. This chronological difference implies a different level of sport experience and performance due to developmental and maturational processes. The aim of the present study was to analyze the relative age effect in elite u-18 basketball according to playing positions. The variables analyzed were: date of birth, playing position and technical-tactical performance indicators in basketball (TTPI). A descriptive analysis was carried out to characterize the sample, a lineal regression was used to analyze the influence of the date of birth on basketball performance and finally an ANOVA and discriminant analysis were performed to identify the differences among different playing positions. The results show the existence of the RAE in the top European Under-18 basketball competition (S1 = 67%; S2 = 33%). Performance indicators which are predictors of the birth quarter (*p*< .05) were only found in the small forwards (defensive rebounds (β: -.463) and fouls received (β: -.140)) and in the centers (three point throws scored (β: -.321) and tried (β: .342)). These results may be of use for coaches and clubs when recruiting players for each playing position. Older forwards and centers are capable of performing at a higher level. Therefore, clubs have to sign up those players born at the beginning of the year.

## Introduction

In the sport context in junior categories, the players are grouped by competition organizers according to their birth date which is how the “sport categories/sport levels” are defined. This format is intended to avoid large age differences among players in the same category. In junior basketball the sport categories are made up of players born in two consecutive calendar years. This means that there may be players competing in the same category with up to two years difference in age. This age difference implies more motor experiences in sport and a greater physical and maturational development which permit the achievement of a better sport performance [1, 2]. The Relative Age Effect (RAE) is the name given to the existence of age differences among individuals in the same category which imply advantages for some players over others due to the greater physical, cognitive and emotional development of those who were born earlier in the competitive year [1–5]. The RAE has been demonstrated to exist in different sport disciplines like ice hockey [3], baseball [4], soccer [5] or basketball [6]. In spite of this, the effects of the RAE have not been clearly explained in specific contexts, like its influence on the initial training stages or after several years of sport participation, competition and training [7]. In basketball, it has been shown that in junior categories, those born in the first half of the year can represent about 80% of players, while in professional categories (the 3 top categories in Spanish basketball) this percentage declines to 60% [8]. Analyzing the 50 all time best players in the NBA, this effect is slightly higher than 50% [8]. In the Olympic Games basketball competition the RAE effect was only found in the French national team [9].

Research on the relationship of physiological and anthropometric characteristics of young players is mainly conducted in soccer [10, 11]. Differences have been found in u-15, u-17 and u-20 categories according to playing position in anthropometry [10, 11] and in physiological performances [11], leading to the conclusion that playing position is characterized by a different profile and specific training sessions should be carried out for each position. Moreover, the main body of research on the RAE and playing position in sports is also focused on soccer [12–14] and handball [15]. In soccer, higher rates of RAE have been found for defenders compared to the other playing positions in male soccer, and between defenders and goalkeepers with midfielders in female soccer. In sports where height and strength are favored, there is an overrepresentation of players born in the first quartiles in positions where larger players have an advantage [15].

Some studies have analyzed the distribution of players according to birth date in basketball, but few have examined how the RAE influences players’ performance [16, 17]. Differences in the performance of players of different ages have been studied in basketball. An analysis of the senior and junior (u-18) world championships showed differences between the expert and novice players [18]. For this study, all performance indicators of junior and senior World Championship were collected. Discriminant analysis highlighted the importance of the greater number of assists and the fewer turnovers of the senior players. Senior players are able read the game situation and context better and make suitable decisions on the spur of the moment, together with having higher technical skills than the juniors have. They, therefore, lose fewer balls and are able to pass the ball to the team mate who is in the best position to score, thus helping team play. The same has been found in junior categories; the u-16 players have better decision-making skills and technical skills than the u-14s [19]. To the best of our knowledge, only one study has analyzed interactive effects of RAE and performance in basketball [16]. This study stated that the older teams perform better, with relatively older male players scoring more point per minute and performance index rating. RAE gradually decreased as the age of players increased. According to playing position, small forwards are the older players, whereas the centers are younger.

Analysis of performance indicators is a consolidated line of research in coaching sciences, providing objective and applicable information for coaches. In basketball, among other studies, the different performance profiles have been analyzed in each of the specific playing positions, point guards, small forwards and centers [20]. The players with different roles have different needs for the competition, confirming that performance indicators both represent how the players interpret the information on the context and how they relate to the others accordingly [21]. For example, it was shown that in the NBA the roles are much more differentiated than in the Spanish ACB league, demonstrating the high specialization of the American competition. In the Portuguese league, the LCB, the point guards and centers have more defensive roles, performing defensive rebounds and blocks, and in the ACB league they stand out for assists and three point throws, while in the NBA they are characterized by offensive rebounds [20].

To our knowledge, no studies have analyzed the impact of the RAE according to the specific playing position in basketball, or the relation of the RAE with the players’ performance evaluated using performance indicators according to playing position. Therefore the purpose of the present study was two-fold: i) examine the distribution of birth dates in competitive basketball in the u-18 category, differentiating by playing position and ii) to analyze the effect of the RAE on performance according to playing position using performance indicators.

## Method

### Sample and variables

The sample was composed of all the players who participated in the Adidas Next Generation Tournament, the top European competition in the u-18 category. A total of thirty-two teams participated per season. The format of the competition was organized in 4 regional tournaments (the Torneig de Bàsquet Junior Ciutat de L'Hospitalet, the Kaunas International Junior Tournament, the Belgrade International Junior Tournament, and the Costa del Sol Tournament). The four champions qualified directly for the Final Four. As well as these teams, the champion from the previous year also qualified directly. The roster was completed by three other teams invited by the tournament organizers, and the event was held in parallel with the Euroleague Final Four. These eight teams competed in two groups and the winning team in each group qualified for the final. All the data were obtained from the official competition webpage: Adidas Next Generation (http://www.adidasngt.com/u18). Data would be found in the specific profile of each player for each season and each tournament. Inclusion criteria are the 2013–14 (http://www.adidasngt.com/u18/game-center/standings?seasoncode=JT13) and 2014–15 seasons (http://www.adidasngt.com/u18/game-center/standings?seasoncode=JT14).

Data were collected from the 2013–14 and 2014–15 seasons (*N* = 767; 100% male) and classified according to the specific position of guards, forwards or centers [20, 22, 23]. The birth years of reference for the 2013–2014 season were 1996 and 1997, and for the 2014–2015 season were 1997 and 1998. In spite of this, the teams sometimes register players who were born in later years (1998 in the first season and 1999 in the second season) and are clearly younger. Such players were excluded from the analysis.

The cut-off date for young categories of clubs participating in Euroleague basketball is the 1^st^ of January (quarters are composed as a function of this cut-off). The variables analyzed include the birth quarter of the players (quarter of the year the players was born: Q1 (January, February, March), Q2 (April, May, June), Q3 (July, August, September) and Q4(October, November, December)), the exact age, the specific position of each one, the minutes played and the following performance indicators: points scored, tried and successful two- and three-point shots, tried and successful free throws, total rebounds, defensive and offensive rebounds, assists, steals, turnovers, blocks committed and received, dunks, personal fouls committed and received and Performance Index Rate (PIR). PIR is calculated according the following formula:

PIR = (points + rebounds + assists + steals + blocks + fouls received)–(missed field goals + missed free throws + turnovers + shots rejected + fouls committed).

Data were collected by the official technicians in each tournament. Nonetheless, data validity was checked using the *multirater κfree index* [24].Two games were randomly selected from each year of competition (four games), analyzed by an expert with previous experience and contrasted with the official data. Kappa index values higher than .91 were obtained for all the variables, except for assists, which obtained a value of .89. According to [25], reliability might be classified as nearly perfect.

### Data analysis

Firstly the performance indicators were normalized and expressed in means per minute of play for each of the players, thus permitting an objective comparison of the sample subjects’ participation. Outliers were cleaned following the procedure stated by Hoaglin and Iglewicz [26]. The distribution of the data was analyzed with the *Kolmogorov-Smirnov* test [27], leading to the selection of the subsequent statistical analyses.

To identify if there was a RAE, an exploratory analysis of the frequencies was carried out to describe the RAE for the whole sample and as a function of the specific playing position in each season studied and the overall players. The distribution of players’ birth dates by quarter as a function of playing position was compared by the chi-square test. Also, odds ratios (ORs) and 95% confidence intervals were calculated for both quartile and half years distribution. In each sample, the number of players reported in each quartile was compared with the expected frequency assuming equal distribution [28]. To identify the effects of the RAE on performance, decimal age was computed. The coefficient of correlation was used to check the correlation between birth dates and performance indicators according to playing position. The statistical analyses were performed using SPSS v.21 software (Inc, Chicago, IL, USA). Statistical significance was set at p < .05.

## Results

Firstly the results are shown of the analysis of the RAE using the frequencies of each of the specific playing positions as a function of the birth quarter (Table 1). In cases where players from the same calendar year revealed a two year age difference with the main age of the category, they were excluded (19 born in 1998; 2 in 1999 and 1 in 2000 in the 2013 season; and 17 born in 1999 and 2 in 2000 in the 2014 season). The final sample was composed of 581 players. As can be seen from the results, in both seasons around 68% of the players were born in the first half of the year; among the guards 72.1% in the first season and 71.7% in the second; in the forwards 66.6% and 71.4% and in the centers 61.8% and 60.9%, respectively. Overall, the first half of the year is overrepresented in the whole sample.

**Table 1:** Descriptive statistics (frequencies and %) as a function of the birth quarter and specific playing position of the players.

		Guards		Forwards		Centers		
		2nd year	1st year	2nd year	1st year	2nd year	1st year	Total
Season 2013–14 1^st^ half of year: 67.9% 2^nd^ half of year: 32.1%	Q1	43(48.3)	23(39.6)	21(32.3)	19(41.3)	14(35.0)	8(22.2)	128(38.3)
	Q2	21(23.5)	19(32.7)	19(29.2)	15(32.6)	13(32.5)	12(33.3)	99(29.6)
	Q3	17(19.1)	9(15.5)	14(21.5)	8(17.3)	8(20.0)	11(30.5)	67(20.0)
	Q4	8(8.9)	7(12.0)	11(16.9)	4(8.6)	5(12.5)	5(13.8)	40(11.9)
		89(26.6)	58(16.7)	65(19.4)	46(13.7)	40(11.9)	36(10.7)	334
Season 2014–15 1^st^ half of year: 68.8% 2^nd^ half of year: 31.2%	Q1	19(33.3)	17(40.4)	19(41.3)	13(28.8)	9(23.0)	8(44.4)	85(34.4)
	Q2	21(36.8)	14(32.5)	15(32.6)	18(40.0)	13(33.3)	4(22.2)	85(34.4)
	Q3	13(22.8)	5(11.9)	4(8.6)	5(11.1)	11(28.2)	4(22.2)	42(17.0)
	Q4	4(7.0)	6(14.2)	8(17.3)	9(20.0)	6(15.3)	2(11.1)	35(14.1)
		57(23.0)	42(17.0)	46(18.6)	45(18.2)	39(15.7)	18(7.2)	247
Overall 1^st^ half of year: 68.3% 2^nd^ half of year: 31.7%	Q1	62(42.4)	40(40.0)	40(36.0)	32(35.1)	23(29.1)	16(29.6)	213(36.6)
	Q2	42(28.7)	33(33.0)	34(30.6)	33(36.2)	26(32.9)	16(29.6)	184(31.6)
	Q3	30(20.5)	14(14.0)	18(16.2)	13(14.2)	19(24.0)	15(27.7)	109(18.7)
	Q4	12(8.2)	13(13.0)	19(17.1)	13(14.2)	11(13.9)	7(12.9)	75(12.9)
		146(25.1)	100(17.2)	111(19.1)	91(15.6)	79(13.5)	54(9.2)	581

Q = Quarter

Table 2 shows the distribution in quarters of the dates of birth of the players according to playing position. Results display a different distribution from expected in both seasons individually and jointly. More players had been born in the first quarters in specific positions of guards and forwards. Centers showed a different distribution, differences were only found when both season were analyzed jointly, whereas when studying both seasons independently birth dates were more evenly distributed.

**Table 2:** Distribution of the dates of birth (percentage) according to playing position for both seasons.

Season	Quarter	Guards		Forwards		Centers	
		Observed	Expected	Observed	Expected	Observed	Expected
2013–2014	Q1	66	36.8	40	27.8	22	19
	Q2	40	36.8	34	27.8	25	19
	Q3	26	36.8	22	27.8	19	19
	Q4	15	36.8	15	27.8	10	19
	χ2	39.585		13.865		6.632	
	Sig.	<0.001		0.003		0.085	
2014–2015	Q1	36	24.8	32	22.8	17	14.3
	Q2	35	24.8	33	22.8	17	14.3
	Q3	18	24.8	9	22.8	15	14.3
	Q4	10	24.8	17	22.8	8	14.3
	χ2	19.990		18.143		3.842	
	Sig.	<0.001		<0.001		0.279	
Overall	Q1	102	61.5	72	50.5	39	33.3
	Q2	75	61.5	67	50.5	42	33.3
	Q3	44	61.5	31	50.5	34	33.3
	Q4	25	61.5	32	50.5	18	33.3
	χ2	56.276		28.851		10.308	
	Sig.	<0.001		<0.001		0.016	

Notes: Q1–Q4 = birth quarter 1–4; χ 2 = Chi-square value; Sig.: significance.

ORs results are presented in Table 3. Based on previous studies [28), the sample revealed significant but small ORs (ranging 1.45 to 3.00) for all comparisons except for guards born in Q1 and Q4 (ORs of 4.08). However, forwards born in Q3 and Q4 did not have a significant relationship (ORs of 0.96).

**Table 3:** Unadjusted odds ratios (ORs) according to player’s position (n) in Adidas Next Generation Tournament examining relative age effect.

	OR comparisons (Q1–4/1st and 2nd 6 months) (95% CI)			
	Q1 vs Q4	Q2 vs Q4	Q3 vs Q4	1st vs 2nd
Guards (246)	4.08 (2.32 to 7.16)	3.00 (1.68 to 5.33)	1.76 (0.96 to 3.22)	2.56(1.76 to 3.73)
Forwards (202)	2.25 (1.27 to 3.98)	2.09 (1.17 to 3.72)	0.96 (0.51 to 1.82)	2.20 (1.46 to 3.31)
Centers (133)	2.16 (1.03 to 4.53)	2.33 (1.12 to 4.85)	1.88 (0.89 to 3.99)	1.55 (0.95 to 2.53)
Total (581)	2.84 (2.00 to 4.02)	2.45 (1.72 to 3.49)	1.45 (1.00 to 2.11)	2.15 (1.69 to 2.73)

The correlation coefficient (Table 4) shows the relation which exists among the performance indicators and the RAE. Performance indicators which are related with the birth date in ANGT (*p*< .05) were found across playing positions. Older guards scored more points and 2-point field goals than those born later. Forward playing positions are related with blocks committed, and centers born early in the year perform and score more 2-point field goals, score more points, receive more blocks and commit more fouls.

**Table 4:** Coefficient of correlation between RAE and performance according to the player’s position.

	Guards	Forwards	Centers
Points Scored	.151*	0.125	.230**
T2 Successful	.132*	0.103	.217*
T2 Tried	0.11	0.108	.214*
T3 Successful	0.107	0.072	0.127
T3 Tried	0.109	0.06	0.066
FT Successful	0.069	0.077	0.044
TT Tried	0.07	0.087	0.092
Total rebounds	0.123	0.082	0.006
Defensive rebounds	0.118	0.062	-0.029
Offensive Rebounds	0.062	0.1	0.046
Assists	0.11	0.106	0.026
Steals	0.104	0.034	0.138
Turnovers	-0.075	0.078	0.049
Blocks committed	-0.032	.198**	0.031
Blocks received	0.068	0.02	.187*
Fouls received	0.039	0.094	-0.17
Fouls committed	0.058	0.093	.199*
PIR	.144*	0.087	.174*
N	246	202	133

*p < 0.05

** p < 0.01

## Discussion

The aims of the present study were to examine the distribution of birth dates in competitive basketball in the u-18 category, differentiating by playing position and ii) to analyze the effect of the RAE on performance according to playing position using performance indicators. The results show the existence of the RAE in the Euroleague ANGT in both seasons, with more players being found to be born during the first 6 months of the year. Furthermore, the analyses show a greater predominance of the RAE in the specific guard position. Performance indicators have also been found which differentiate the actions carried out by the players according to their playing position according to birth date.

The RAE has often been identified in different sports, with differing effects according to sport discipline or type of competition. The evidence suggests that, although this phenomenon exists, its effect tends to disappears when the level increases [6, 7, 16] and does not predict a high performance career or selection for national teams [9]. In fact, the youngest players enjoy longer sport careers [29]. In this study, there was an overrepresentation of players born in the first six months of the year. In basketball, young players perform better according to anthropometrics such as height [17, 30, 31], or physiological characteristics [17]. Players born early in the year have an advantage in these characteristics [6, 32]. Coaches and clubs use criteria based on greater physical and anthropometric development when selecting players to participate in these competitions, resulting in a biased distribution of birth dates. This practice should be questioned as far as is possible, as it is considered that birth date should be taken into consideration when selecting and developing young talents [33]. Ibáñez, Sáenz-López [34] identified a negative correlation in the participation of players in national junior teams, and their subsequent development and participation in professional leagues and senior national teams. The RAE has different implications in players´ development. Those athletes born earlier in the year have more motor experiences in the sport context, leading to a better sport performance than their pairs born late in the year [2, 35]. Also, older players could benefit from more skilled coaches, better facilities and sport programs and higher competition levels [36–38]. On the other hand, younger players have to overcome their chronological limitations; having less motor experiences leading them to develop skills to compete with their pairs [17]. These skills will permit them to achieve better sport performance in adulthood, since the advantages based on birth date disappear and will no longer be present [17].

In basketball, it is common for guards to be smaller and shorter than the rest of the team. They are the players that organize the team’s offensive play, deciding who should have the ball at any one time; therefore needing a good vision of the court and good passing skills. Finally, guards play most of the time away from the rim and, consequently, they have to develop good field-goal shooting. Centers are usually the tallest players in the team, playing near the rim. Thus, these players need a large body mass to score and defend close to the basket. Finally, forwards are the players with intermediate stature, who have to have good field-goal shooting skills to score away from the rim, and need good conditioning and fitness to help near the basket to rebound and score [39]. In junior or sub elite categories, the physical differences are not as great as in elite basketball. In fact, no significant differences have been found among specific positions in weight, height or anaerobic capacity in u-18 basketball, but they have been found in total relative strength [40]. As stated before, older players have greater body mass, size and strength which means that they perform better [41], confirming the beliefs of the coaches [42].

The RAE is more visible in guards than in any other position (ORs and percentages), whereas centers are the players in whom the RAE is less evident. Guards that train more and have greater competitive experience, acquire their performance skills faster than others playing positions, thus height or size is not essential for this position. In contrast, forwards and centers depend more on height and weight to achieve a high performance and participate in elite sport. These qualities are innate and are not attained with training. In university basketball the players who develop as point guards are more important in the game, in contrast to the centers who, because they have to grow taller, take longer to fully develop [43]. Although speculative, it is possible that this is one of the reasons for the predominance of the RAE in guards, as their continuity in this sport activity at the high level depends more on their sport skills and abilities than on their anthropometric measurements.

The RAE also influences performance in playing positions. Statistical analysis shows a relation between guards and points scored, successful 2-point field-goals and PIR, forwards and blocks committed, and centers and points scored, successful and unsuccessful 2-point field-goals, blocks received, fouls committed and PIR. Players with more experience, such as older ones [35], are able to better interpret the information from the context as they have more developed attention and selection processes [44]. This means that they are capable of better perceiving the information from the context and anticipating the game before their opponents and, for example, have a better position for blocking and helping in defense. Also, these attention processes, along with better technical skills [44] and offensive strategies [45] allow older players, guards and centers, to make better shot selections and achieve more points scored and successful 2-point field goals and overall PIR. Also, in basketball it has been stated that rebounds and 2-point field goals are the more discriminant performance indicators for winning a game [21], and are primarily performed by centers [20]. The older players in the center position committed more fouls and received more blocks than the younger ones. Older more experienced players, have better attention processes and offensive strategies [44, 45]. These centers are capable of being aware of the needs of the game and play more assertively, taking risks at different moments and, thus, exposing themselves to mistakes such as fouls committed and blocks received, actions with more probabilities of happening near the basket.

## Limitations

Some limitations have to be addressed. The maturity status has been proved important in player selection processes. Although the growth process is near to finishing or supposedly finished in this sample (u-18), the study helps to clarify and complete the conclusions on the RAE and players’ development in basketball.

## Conclusions and practical applications

The RAE exists in the most important junior continental basketball competition, the Adidas Next Generation Tournament. Guard is the playing position that presents more probabilities of been born in the first half of the year, whereas center is the position that presents less. This way of selecting players is based on advantages related with birth dates; advantages that are smoothed out or eliminated when players achieve full maturation. Also, the qualities necessaries to achieve top level performance as adults do not appear until late adolescence. Results confirm that players’ selection processes are biased negatively for players born at the end of the year. Those players, that have the potential to perform basketball at a high level, are denied access to the best training processes and coaches, hence losing their chances to reach their potential.

The results show the need to re-direct talent development processes in basketball. Coaches should consider these results, the RAE should not be overestimated in player selection processes. New criteria should be used to select the talented players, combining their current performance with their potential future performance. Clubs and federations should have development programs that consider that RAE is not decisive for being a high performance basketball player. It has also been shown that birth date affects the playing profile for each playing position. Coaches should use this information to create training and improvement programs which are more suitable and specific for each profile, enhancing the contribution of each player to the overall performance of the team.
